# Editorial: Urothelial carcinoma of renal pelvis and ureter, prognosis and recent advances

**DOI:** 10.3389/fsurg.2023.1228589

**Published:** 2023-08-17

**Authors:** Shu-Yu Wu, Yao-Chou Tsai

**Affiliations:** ^1^ Department of Urology, Taipei Tzu Chi Hospital, Buddhist Tzu Chi Medical Foundation, New Taipei City, Taiwan; ^2^ School of Medicine, Buddhist Tzu Chi University, Hualien, Taiwan

**Keywords:** UTUC (renal pelvis & ureter), radical nephrectomy, radical nephroureterectomy (RNU), elderly, urothelial cancer (UC)

**Editorial on the Research Topic** Urothelial carcinoma of renal pelvis and ureter, prognosis and recent advances

## Introduction

Urothelial carcinoma of the renal pelvis and ureter is a prevalent malignancy primarily affecting the elderly population (1). Its incidence has been on the rise in recent years (2). With evolving knowledge of the disease, significant advancements in prognosis prediction and treatment strategies have emerged. This editorial article aims to discuss the contributions of recent studies in this special issue regarding UTUC, shedding light on prognosis and introducing novel approaches to improve patient outcomes.

### Insights into mortality patterns and therapeutic strategies

A comprehensive analysis exploring the causes of death among upper tract urothelial carcinoma (UTUC) survivors has provided valuable insights into mortality patterns. By utilizing the National Cancer Institute's Surveillance, Epidemiology, and End Results (SEER) database, a large cohort of UTUC patients was examined (Zhanghuang et al.). The study revealed that non-UTUC deaths accounted for the majority of mortality among patients with localized disease. This highlights the importance of considering non-cancer causes in the management of UTUC survivors. Moreover, UTUC was found to be the leading cause of death in patients with regional and distant stages, emphasizing the need for improved therapeutic strategies targeting advanced disease while considering the increased risk of death from non-cancer causes (2).

### Significance of surgical methods and precision intervention

A propensity score-matched study assessed the impact of surgical methods on overall survival (OS) and cancer-specific survival (CSS) in renal pelvic urothelial carcinoma (RPUC) patients. Using data from the SEER database, the study compared radical nephroureterectomy (NU) and inadvertent radical nephrectomy (RN). The results underscored the significance of accurate diagnosis and appropriate surgical intervention in RPUC patients. The study concluded that RN could lead to worse oncological outcomes compared to NU, emphasizing the importance of precise surgical planning and execution.

### Differentiating radical nephroureterectomy and inadvertent radical nephrectomy

Another study emphasized the importance of distinguishing between radical nephroureterectomy (NU) and inadvertent radical nephrectomy (RN) in patients with renal pelvis urothelial carcinoma (RPUC). A retrospective analysis of data from the SEER database revealed that patients who underwent RN experienced worse overall survival (OS) compared to those who received NU. Furthermore, the study highlighted that the negative impact of RN on OS was more significant in patients with tumors larger than 4.2 cm (Wu et al.).

### Exploring surgical approaches and predictive factors

A study utilizing data from the Taiwan nationwide upper urinary tract urothelial carcinoma (UTUC) collaboration database evaluated the outcomes of transperitoneal hand-assisted laparoscopic nephroureterectomy (TP-HALNU) and transperitoneal pure laparoscopic nephroureterectomy (TP-LNU) (Kuo et al.). While no significant differences were observed in surgical outcomes between the two approaches, the study identified various predictive factors associated with adverse oncological outcomes. Age over 70, positive lymph node metastasis, upper ureter tumor location, and male sex were identified as potential risk factors. Additionally, higher surgical volume showed trends toward favorable outcomes in terms of overall survival and cancer-specific survival.

### Role of nephroureterectomy in stage IV UTUC

A multicenter retrospective cohort study addressed the role of nephroureterectomy (NU) in stage IV upper tract urothelial carcinoma (UTUC) (Lin et al.). The findings indicate that patients who received chemotherapy (CT) combined with NU exhibited improved overall survival (OS) compared to those who received CT alone. This benefit was observed in both nonmetastatic and metastatic UTUC. The study emphasizes the potential advantages of NU in prolonging survival outcomes for patients with advanced-stage UTUC.

## Conclusion

Collectively, these studies contribute to our understanding of urothelial carcinoma of the renal pelvis and ureter, highlighting the significance of accurate diagnosis and appropriate surgical management. Inadvertent radical nephrectomy (RN) was shown to have a negative impact on overall survival (OS), particularly in patients with larger tumors. Furthermore, while the choice between surgical approaches may not significantly influence outcomes, other factors such as patient age, lymph node metastasis, tumor location, and surgical volume should be considered for prognostic evaluation. Finally, the role of nephroureterectomy (NU) in stage IV UTUC demonstrates potential benefits in terms of overall survival (OS) for both nonmetastatic and metastatic cases. These findings underscore the importance of precise diagnosis and treatment among patients with UTUC.
